# Community *Legionella* outbreak linked to a cooling tower, 2022

**DOI:** 10.14745/ccdr.v49i09a04

**Published:** 2023-09-01

**Authors:** Steven Rebellato, Colin Lee, Charles Gardner, Karen Kivilahti, Jenee Wallace, Danielle Hachborn, Jillian Fenik, Anna Majury, JinHee Kim, Allana Murphy, John Minnery

**Affiliations:** 1 Simcoe-Muskoka District Health Unit, Barrie, ON; 2 Public Health Ontario, Toronto, ON

**Keywords:** *Legionella*, outbreak, cooling tower, Ontario, Canada

## Abstract

**Background:**

Thirty-five laboratory-confirmed legionellosis cases were reported to the Simcoe Muskoka District Health Unit (Ontario, Canada) between September 27, 2022, and October 15, 2022, resulting in one death and 29 hospitalizations. This article describes the *Legionella* outbreak and highlights activities for managing the outbreak, including various environmental and infrastructural controls associated with the public health response and some of the unique challenges and potential solutions to mitigate future outbreaks.

**Methods:**

All cases of legionellosis were reported to and investigated by the local provincial health unit. Within a 6 km radius around the community, 27 cooling towers (CTs) were identified as potential sources of *Legionella*. Environmental samples were collected from 19 CTs and a long-term care home.

**Outcome:**

Of the 35 cases, 29 (83%) were hospitalized (including three long-term care residents) with two requiring intubation/ventilation. Of the five sputa (clinical isolates) collected from confirmed cases, four tested positive for *Legionella pneumophila* (one was positive for *L. pneumophila* serogroup 1—with the same sequence type as one of the CT isolates). Education and recommendations were provided by the local provincial health unit to operators to improve CT operation.

**Conclusion:**

Detection and management of community legionellosis outbreaks associated with CTs involve resources and time to properly identify and control risks. Measures for community risk mitigation included coordinating with provincial and community partners, developing methods to rapidly identify CTs as a likely source of infection and applying operational/maintenance/testing standards for CTs to control bacterial growth and minimize the dispersion of contaminated aerosols.

## Introduction

Legionellosis is caused by the *Legionella* bacteria; most commonly *Legionella pneumophila* ((1)). While the bacteria are commonly found in natural, freshwater environments, it can become a health concern in human-made water systems (e.g. plumbing systems of large buildings, cooling towers [CT], certain medical devices, decorative fountains) where conditions allow it to multiply. People contract *Legionella* by inhaling aerosolized water droplets containing the bacteria or, less commonly, by aspiration of contaminated drinking water. Legionellosis may present as Legionnaires’ Disease or Pontiac Fever with symptoms including anorexia, malaise, myalgia, headache, productive cough, fever, pneumonia, confusion, chills, nausea and diarrhea ((1)). Ontario provincial data ((2)) reported a rise in legionellosis incidence from 2012 (rate=1.4/100,000; 191 cases) through 2021 (rate=2.6/100,000; 385 cases) with individuals 50 years of age or older representing most reported cases. Legionellosis is a disease of public health significance under the Government of Ontario’s *Health Protection and Promotion Act* ((3)) and is reportable to the local provincial health unit where the individual resides. No province-wide regulatory framework associated with inventory, design, maintenance, operation or testing of CTs for the purpose of mitigating *Legionella* outbreaks exists in the province of Ontario as observed in some other Canadian jurisdictions ((4–6)). However, following the 2019 community outbreak, a provincial public health association recommended developing a CT registry, in addition to risk management and maintenance plans for owners and operators ((7)). As of the date of this publication, no provincial CT registry exists in the province of Ontario.

Thirty-five laboratory-confirmed cases of legionellosis were reported to the Simcoe-Muskoka District Health Unit (Ontario, Canada) between September 27, 2022, and October 15, 2022. An outbreak of legionellosis in the same community in 2019 resulted in 10 cases (all admitted to hospital) and one death. A CT was linked by laboratory testing with one of the cases. Given the 2019 outbreak, and recognizing that CTs are the most frequent source of large community outbreaks ((8–12)), CTs were investigated in 2022 as a source.

This report describes a community outbreak of legionellosis in the fall of 2022 in Ontario. The article focuses on the epidemiology and investigative processes leading to the outbreak’s conclusion. The article highlights the continued challenges that public health practitioners experience in the identification and control of *Legionella* in relation to CT units and how a registry could facilitate rapid identification of CTs in a region of interest, as well as how guidance and/or regulations could support ongoing maintenance and monitoring as preventive public health measures.

## Methods

### Case investigation

The first confirmed case of legionellosis was reported to the health unit on September 27, 2022. On October 4, 2022, four cases of legionellosis were admitted to the local hospital, presenting with pneumonia. Analysis of case movement and exposures revealed that all four cases resided or worked in the same community based on postal code. Given the monthly expected case counts, based on the five-year mean for the public health region is between one and three cases in September and October, the observed case count (n=4) in a single community was aberrant. Accordingly, an outbreak was declared by the health unit on October 4, 2022. An outbreak case was defined as any individual who lived, worked or visited the identified community in Simcoe County, with compatible signs and symptoms of legionellosis, on or after September 5, 2022, and who had laboratory confirmation of legionellosis. The outbreak investigation was initiated on October 4, 2022. Using the seven-day median reporting time from symptom onset plus the maximum incubation period of 15 days, in addition to allowing for two additional days of coordinating communications, the outbreak was declared over on November 8, 2022, which represented 24 days from the last reported case (October 15, 2022). The outbreak lasted 35 days.

Urine specimens were submitted to the Public Health Ontario Laboratory. Urinary antigen tests were performed by the laboratory for all suspect cases. Upon report of a new case, the health unit conducted a case interview and exposure history that included the 14 days prior to symptom onset, identifying home location, work location and any other locations visited during this period. An exposure associated with this outbreak was defined as any location within the area of investigation, where the case spent any amount of time during their period of acquisition (e.g. workplace, home, shopping, day excursion).

### Interventions and environmental investigation

Given the hospitalization and laboratory confirmation of five cases of legionellosis with pneumonia at the local hospital from September 27 to October 4, 2022, the health unit initiated an investigation into potential environmental sources. Community and healthcare providers were informed of the outbreak to assist in facilitating case finding, prompt diagnosis and management of persons with potential legionellosis. The public was notified of the outbreak via local media to 1) inform those who may be at risk, 2) describe clinical signs and symptoms, and 3) recommend that those who are symptomatic seek medical attention if their symptoms are severe or do not resolve.

A review of case investigation data revealed that no single exposure site or activity was common among the five cases, other than travelling to, living or working in the community. A line list of cases with exposures was developed and, on review, no common exposures for the cases were identified other than geography suggesting an environmental exposure covering an area defined by a 6 km radius. Through elimination of other potential sources, including municipal drinking water, CTs were identified as the probable source. On October 5, 2022, 15 CTs were identified within the 6 km radius using location data collected during the 2019 outbreak. A 6 km radius was established based on previous literature ((13)) that described this radius as a reasonable distance for aerosol dispersion. The health unit requested operators of these known CTs to immediately cease operation unless deemed by the operator as essential to the facility’s operation. The CTs that remained operational were required to provide maintenance and testing records from July to October 2022 and directed to arrange for immediate cleaning and disinfection.

The owners/operators for 11 of 15 CTs provided monitoring data collected through routine *Legionella* maintenance programs. All quantitative *Legionella* culture results were reported as fewer than 10 colony forming units (CFU)/mL for *Legionella* species. According to the Canadian guidelines for federal buildings ((14)), a value of fewer than 10 *Legionella* spp. CFU/mL is considered within the acceptable limit. Laboratory data for the remaining four CTs were not available from the CT owner/operator as no sampling program was in place. From October 8 to November 1, 2022, public health inspectors identified additional CTs (total n=27) and collected water and biofilm samples from 19 CTs for submission to the Public Health Ontario Laboratory. Eight other CTs could not be sampled due to seasonal shut down. During the investigation, one operator/owner was ordered to shut down their CT due to unsanitary conditions, lack of routine maintenance and lack of testing. The CT previously identified as the source of the 2019 outbreak could not be immediately sampled by public health officials due to a mechanical failure that rendered it devoid of water.

On October 17, 2022, the health unit received culture results from a CT showing *L. pneumophila* serogroup 1 levels of 2,575 CFU/mL. The sample was collected on September 27 and tested as part of routine monthly testing. The health unit arranged to have the September 27 isolate transported to the Public Health Ontario Laboratory for sequence-based typing (SBT). Sequence-based typing was performed to determine the relatedness of the clinical and environmental isolates. The SBT method at Public Health Ontario Laboratory is based on the epidemiological typing scheme for clinical and environmental isolates of *L. pneumophila*, which was developed by members of the European Legionnaires’ Disease Surveillance Network and evaluated for implementation in the investigation of outbreaks of legionellosis caused by *L. pneumophila*. Culture results can take up to 14 days to be received, which can delay interventions. In this case, no other immediate action was required given the CT had already been shut down on October 7 due to mechanical failures. Between October 17 and November 10, the health unit also conducted quantitative polymerase chain reaction (qPCR) testing using field equipment to quantify results for further decision-making. The Federal Standard guided the corrective actions taken and the interpretation of results. The CTs selected for qPCR testing were prioritized based on 1) lack of maintenance and testing, 2) missing test results or 3) requirement to further evaluate samples from those CTs where qPCR and culture showed the presence of *Legionella* spp.

A separate environmental investigation took place for a sub-cluster of cases identified at a long-term care home (LTCH) within the 6 km radius. Three residents of the LTCH were hospitalized and determined to be *L. pneumophila* serogroup 1-positive based on urinary antigen tests. Due to the case investigation, two shower rooms were identified as potential sources and sampled on October 8, 2022. All samples yielded negative results by culture.

### Epidemiologic and statistical analyses

**Figure 1:**
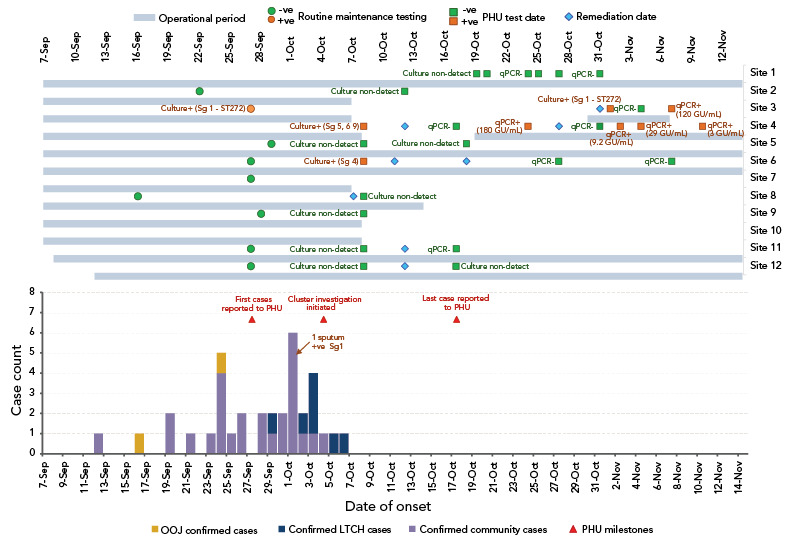
Environmental testing and epidemiological curve including key milestones during cluster investigation Abbreviations: LTCH, long-term care home; OOJ, out of jurisdiction; PHU, public health unit; qPCR, quantitative polymerase chain reaction; Sg, serogroup; +ve, positive; -ve, negative Data sources: Investigation Line List and Environmental Health Sample Collection spreadsheet

Descriptive epidemiology was used to enumerate total cases, the severity of cases and their outcomes. An epidemic curve was created in Microsoft Excel (**Figure 1**). It displays cases over time, with differentiation between community cases, cases residing in a LTCH, and cases from out of jurisdiction of the local health unit. The environmental investigation results of CTs are displayed above the cases over the same time scale.

To explore sources of disease, ESRI ArcGIS Desktop^®^ 10.6.1 was used to map cases by home location and assess clustering in investigation, followed by mapping of cases’ exposure locations and 27 known CTs identified during the outbreak period (and the testing results, as available). A hotspot analysis was undertaken using the Optimized Hot Spot Analysis Spatial Statistic Tool using a 6 km radius buffer around the area of investigation and a cell size of 200 m.

Hotspot analysis and exposure history analysis were rerun as new confirmed cases were identified and the outbreak progressed. Upon identification of a sequence type (ST) match between the isolate cultured from a case’s sputum and an isolate cultured from the implicated CT, assessment of each case’s nearest exposure to the CT was conducted.

## Results

Thirty-five laboratory-confirmed cases were identified in this outbreak and were associated with having a residence and/or having visited locations within a 6 km radius of a community within the jurisdictional area of the local health unit. Of the 35 laboratory confirmed cases, 29 were hospitalized. One case died, resulting in a case-fatality rate of 2.9%. Twenty-six cases resided in the community, seven cases were LTCH residents, and two cases were residents of other communities who visited the area during their acquisition period. **Table 1** shows a breakdown of cases by demographics (age, sex, location), health conditions and severity. Twenty-nine of the 35 cases (83%) were hospitalized, including three of the LTCH residents, with two hospitalized cases admitted to the intensive care unit requiring intubation/ventilation.

**Table 1:** Characteristics of confirmed Legionellosis cases included in the outbreak investigation

Characteristics of cases	Total	
	N	%
Total cases	35	100%
**Sex**		
Female	17	48.6%
Male	18	51.4%
**Age group (years)**		
50–59	7	20.0%
60–69	12	34.3%
70–79	5	14.3%
80 and older	11	31.4%
**Residential location**		
Local community	26	74.3%
Long-term care home	7	20.0%
Out of jurisdiction	2	5.7%
**Chronic conditions**		
Any	27	77.1%
None	2	5.7%
Unknown	6	17.1%
**Hospitalization status**		
Not hospitalized	6	17.1%
Hospitalized	27	77.1%
Intensive care unit	2	5.7%
**Outcome**		
Recovered	34	97.1%
Death	1	2.9%

Of the five sputa collected from laboratory-confirmed cases, four specimens were found to be positive for *L. pneumophila* via polymerase chain reaction (PCR), with one of the five also having a positive culture for *L. pneumophila* serogroup 1. Given that culture isolates were available for both a clinical and an environmental source, SBT followed.

Of the 27 CTs identified in the 6 km radius zone, environmental samples were collected from 19 CTs and 2 samples from plumbing within the involved LTCH. *Legionella* spp. was not detected in any of the samples collected from the LTCH. Of the 19 CTs, eight had *Legionella* spp. detected by PCR (which detects DNA from both viable and non-viable *Legionella*), three of which were identified as *L. pneumophila* serogroup 1. *Legionella pneumophila* serogroup 1 was isolated by culture from only one of the CTs. Subsequent SBT of the *L. pneumophila* serogroup 1 isolate was genetically similar to the isolate from the case’s sputum, as both were identified as ST 272.

### Mapping and hotspot analysis

**Figure 2:**
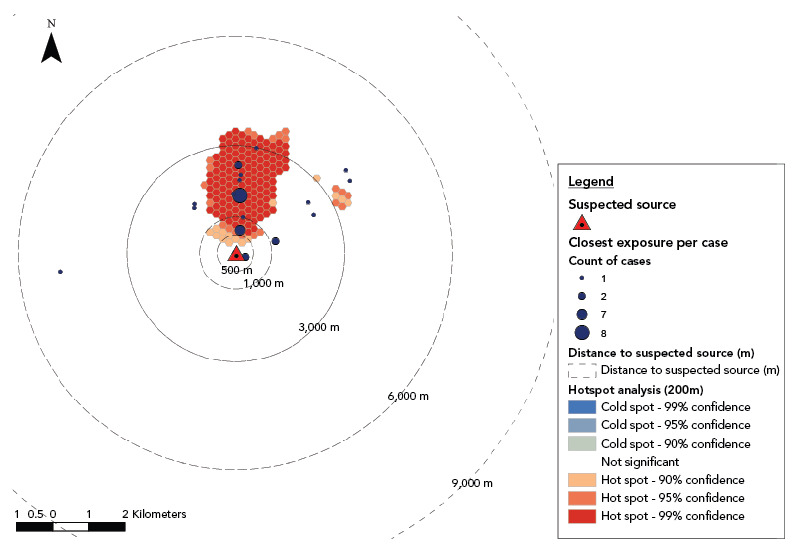
Closest case exposure locations in relation to suspected source with hotspot analysis Abbreviations: m, metres; qPCR, quantitative polymerase chain reaction Source: Legionellosis Cluster 2022 Investigation Case Data and Environmental Health Data

The hotspot analysis of case exposure locations identified two clusters: a large (approximately 2 km^2^) cluster in the north-west of the investigation area and a smaller 600 m^2^ cluster in the centre of the area (**Figure 2**). Samples from the CT from which *Legionella* was isolated had the same ST as the *Legionella* isolated from the case and was located at the southern end of the large cluster. All cases had at least one exposure within a 6 km buffer from the subject CT:

• 2 cases were onsite

• 2 cases within 500 m

• 7 cases between 500 m and 1 km

• 21 cases between 1 km and 3 km

• 3 cases between 3 km and 6 km

## Discussion

Thirty-five laboratory-confirmed cases of legionellosis were investigated between September 27, 2022, and October 15, 2022. Through epidemiological and geospatial/hotspot analysis of case exposure locations, it was hypothesized that a community source(s) was responsible for the outbreak since no common single location/facility was identified amongst case histories for the majority of cases. Furthermore, the approximately 2 km^2^ cluster in the north-west area and smaller 600 m^2^ cluster in the centre of the area of the community (Figure 2) contain several large retail stores that were common destinations amongst the cases.

In addition to urine specimens being submitted for testing, sputum specimens were submitted for five cases, one of which was positive for *L. pneumophila* serogroup 1 by culture. Of the 19 CTs investigated and tested for *Legionella*, *L. pneumophila* serogroup 1 was cultured from one CT. Using SBT, it was determined that the isolate from the CT and the clinical isolate were the same ST. Despite remediation of the CT being identified as the outbreak source, subsequent testing for *Legionella* continued to yield positive results of *L. pneumophila* via qPCR, PCR and culture. Thus, the CT was shut down indefinitely on November 6, 2022. Following two incubation periods with no reported cases in the defined geographical area, the investigation was concluded and the public was notified that the outbreak was declared over.

This outbreak highlights the need to rapidly identify CTs during public health investigations to assess their design, maintenance and operation from the *Legionella* risk mitigation perspective. Seventeen days had elapsed by the time all CTs within the 6 km radius were identified and confirmed following the declaration of the outbreak on October 4, 2022. Should a registration system been in place, it is likely that CTs would have been accessed and investigated in an accelerated manner. Further, multiple outbreaks within the same community within a three-year period reinforce the need for CT monitoring, maintenance, risk assessment and risk management—which did not occur in a number of systems that were previously investigated. The outbreak also highlights the value of coordinated environmental and clinical sampling to assist in source identification using molecular typing strategies and to support effective public health action. Frequent and timely availability of quantitative *Legionella* monitoring may serve as an important adjunct to routine maintenance, particularly if adverse findings are reportable as an indicator of the effectiveness of maintenance and remediation practises. Moreover, even in the absence of *Legionella* cases being identified in the community, routine *Legionella* monitoring would result in increased awareness by owners and operators of the risks and expectations and should help mitigate future outbreaks given adverse results would prompt immediate remediation practises, acting as an early warning system.

The outbreak epidemiology and investigation results were similar in scope to a community outbreak reported in Montréal, Québec in 2019 ((1)). Both investigations involved intensive public health investigation resources (extensive environmental sampling, coordination, communication) along with the challenges of seeking a clinical-environmental link, including the need for additional clinical isolates for typing to assist in the investigation process. With several similarities between outbreaks, there were some notable differences. In particular, the province of Québec already requires CTs to be registered and have maintenance programs in place ((8)). While it could be argued that the Québec outbreak puts into question those jurisdictions with existing registries for CTs, the study concluded that the province of Québec would benefit from further development of provincial registries for other water aerosolization sources given the potential of CTs for transmission of *Legionella* and the inability of the outbreak investigation to identify a source.

## Challenges

Several challenges were identified throughout this outbreak investigation. Of the 35 cases, only one of the five clinical sputum specimens submitted yielded a culture isolate, which is required to perform SBT. Providers had often started appropriate antibiotic treatment for hospitalized patients with pneumonia before receiving a positive urinary antigen test. Respiratory specimens for culture were not usually taken after those urinary antigen test results, as clinicians do not require test results from sputum specimens for further antimicrobial management of those patients. Moreover, those patients may have already been discharged. In this outbreak, SBT of a clinical isolate was genetically similar to an isolate cultured from a single CT sample. In addition to a lack of clinical specimens, not all CTs in the identified 6 km radius could be sampled and tested given some were not operational at the time sampling was being undertaken, despite being in operation during the incubation period. Furthermore, many facilities do not participate in a routine *Legionella* monitoring program; thus, historical data were not available for retrospective review to assess which CTs may have been highest risk. An ongoing challenge for public health in lieu of an accessible community or provincial CT registry is the identification of CTs during an outbreak situation and the requirement for CTs to be maintained and sampled on a routine basis. If a registry and requirement for sampling and maintenance existed, it would allow for accelerated communication and appropriate analysis of sampling records to take place to focus on higher-risk systems. Particularly for systems within an identified geographical area of interest based on case analysis for suspect community *Legionella* outbreaks, historical sampling data for suspected systems would assist in determining trends in seasonality, operational periods, and in turn, provide the opportunity for education and intervention in seeking optimal system operation. Finally, hotspot analysis was simplified and did not account for some cases having multiple exposures in a small geographical area.

## Conclusion

This community outbreak resulted in 35 laboratory-confirmed cases of legionellosis. Using sequence-based typing, it was determined that one environmental isolate from a CT and one clinical isolate were the same (relatively uncommon) ST.

This report describes the challenges of managing a coordinated clinical and environmental investigation of a community *Legionella* outbreak in Ontario and reiterates the broader public health risks posed by the organism in CTs. The detection and management of community *Legionella* outbreaks associated with CTs is complex. Coordination with provincial and community partners is critical to the investigation process. Challenges in other jurisdictions similar to the one described here have resulted in the introduction of universal CT registries, which have facilitated the identification of community CTs during a rapid public health response. Moreover, some responses have included the implementation of CT monitoring programs that include *Legionella* testing at regular intervals, with prompt and mandatory reporting requirements to public health authorities, to proactively identify potential *Legionella* sources. Ultimately, what is essential is appropriate management and maintenance programs, with oversight from qualified water quality personnel for optimal operation of CTs, reduced bacterial growth and the associated public health risks.
